# Oral Microbiome Analysis in Prospective Genome Cohort Studies of the Tohoku Medical Megabank Project

**DOI:** 10.3389/fcimb.2020.604596

**Published:** 2021-01-29

**Authors:** Sakae Saito, Yuichi Aoki, Toru Tamahara, Maki Goto, Hiroyuki Matsui, Junko Kawashima, Inaho Danjoh, Atsushi Hozawa, Shinichi Kuriyama, Yoichi Suzuki, Nobuo Fuse, Shigeo Kure, Riu Yamashita, Osamu Tanabe, Naoko Minegishi, Kengo Kinoshita, Akito Tsuboi, Ritsuko Shimizu, Masayuki Yamamoto

**Affiliations:** ^1^Tohoku Medical Megabank Organization, Tohoku University, Sendai, Japan; ^2^Graduate School of Information Sciences, Tohoku University, Sendai, Japan; ^3^Graduate School of Dentistry, Tohoku University, Sendai, Japan; ^4^Graduate School of Medicine, Tohoku University, Sendai, Japan; ^5^International Research Institute of Disaster Science, Tohoku University, Sendai, Japan; ^6^Department of Clinical Genetics, Ageo Central General Hospital, Ageo, Japan; ^7^Exploratory Oncology Research & Clinical Trial Center, National Cancer Center, Kashiwa, Japan; ^8^Biosample Research Center, Radiation Effects Research Foundation, Hiroshima, Japan; ^9^Advanced Research Center for Innovations in Next-Generation Medicine, Tohoku University, Sendai, Japan

**Keywords:** 16S ribosomal RNA, saliva, dental plaque, microbiome, periodontal disease, prospective cohort, oral health

## Abstract

A baseline oral microbiome study of the Tohoku Medical Megabank Organization (TMM) was planned to characterize the profile of the oral microbiome in the Japanese population. The study also aimed to clarify risk factors for multifactorial diseases by integrated analysis of the oral microbiome and host genome/omics information. From 2013 to 2016, we collected three types of oral biospecimens, saliva, supragingival plaque, and tongue swab, from a total of 25,101 participants who had a dental examination in TMM. In this study, we used two independent cohorts; the Community-Based Cohort and Birth and Three-Generation Cohort as discovery and validation cohorts, respectively, and we selected participants examined by a single dentist. We found through the 16S ribosomal RNA gene sequencing analysis of 834 participants of the Community-Based Cohort Study that there are differences in the microbial composition and community structure between saliva and plaque. The species diversities in both saliva and plaque were increased in correlation with the severity of periodontal disease. These results were nicely reproduced in the analysis of 455 participants of the Birth and Three-Generation Cohort Study. In addition, strong positive and negative associations of microbial taxa in both plaque and saliva with periodontitis-associated biofilm formation were detected by co-occurrence network analysis. The classes *Actinobacteria* and *Bacilli*, including oral health-associated bacterial species, showed a positive correlation in saliva. These results revealed differences in microbial composition and community structure between saliva and plaque and a correlation between microbial species and the severity of periodontal disease. We expect that the large database of the oral microbiome in the TMM biobank will help in the discovery of novel targets for the treatment and prevention of oral diseases, as well as for the discovery of therapeutic and/or preventive targets of systemic diseases.

## Introduction

A prospective genome cohort study with long-term observational data for large healthy populations is a key activity to elucidate the pathogenesis of multigenic and multifactorial diseases (Donnelly, 2008; McCarthy et al., 2008; Plomin et al., 2009). Multifactorial diseases developed by an interaction between genetic and environmental factors are common in the general population and account for many chronic diseases (Plomin et al., 2009). The Tohoku Medical Megabank (TMM) project was established to conduct large-scale prospective genome cohort studies in Miyagi and Iwate Prefectures after devastating damage resulted from the Great East Japan Earthquake in 2011. The project was conducted by the Tohoku Medical Megabank Organization (ToMMo) and Iwate Tohoku Medical Megabank Organization (IMM). The aim of this project is to contribute to the health maintenance of residents in the tsunami devastated area and to form a solid foundation of personalized healthcare by strategically constructing two prospective genome cohorts and an integrated biobank (Fuse et al., 2019).

We are conducting two prospective genome cohort studies in the TMM project (Kuriyama et al., 2016). One is a population-based cohort study called the TMM Community-Based Cohort Study (TMM CommCohort Study) targeting adult individuals (aged 20 and older), and this study recruited a total of 87,865 participants from 2013 to 2016 (Hozawa et al., 2020). The other is a birth and three-generation cohort study called the TMM Birth and Three-Generation Cohort Study (TMM BirThree Cohort Study), which has a family-based prospective study design, including newborns and their parents, siblings, grandparents, and extended family members. In the TMM BirThree Cohort Study, we recruited 73,529 participants, including 22,493 mothers and 23,143 newborn children, from 2013 to 2017 (Kuriyama et al., 2019).

In the TMM biobank, all biospecimens and clinical information collected from cohort participants have been registered and rendered for systematic storage. The TMM biobank aims to be an integrated biobank in which ordinal analyses are conducted by an analytical center affiliated with the biobank. At first, data are shared by the users, and if samples are still necessary, the biobank shares biological samples. Therefore, multiomics analyses, such as genomics, transcriptomics, epigenomics, proteomics and metabolomics, have been performed using these biospecimen resources in the biobank (Takai-Igarashi et al., 2017; Minegishi et al., 2019). By 2019, ToMMo performed whole-genome sequencing of over 80,000 participants and constructed a Japanese whole-genome reference panel that provides information on the frequency of genetic variations in the Japanese population (Tadaka et al., 2018; Yasuda et al., 2019).

Based on the Japanese genome reference panel data, we designed a custom single nucleotide polymorphism (SNP) array aiming to conveniently collect the SNP genotype data for all cohort participants by means of microarray technology. ToMMo has also been conducting a comprehensive metabolome analysis of blood plasma and has finished the analysis of approximately 15,000 participants (Koshiba et al., 2018). The multiomics data analyzed in the TMM project are available in a public database, the Japanese Multi Omics Reference Panel (jMorp), which provides comprehensive information on human life sciences (Tadaka et al., 2018).

As part of the TMM cohort study, we planned to clarify how symbiotic microbiota influences our health and wellness. Therefore, a large-scale human microbiome analysis was conducted to identify profiles of symbiotic microbiota, which are environmental factors in the human body. The human microbiome, which is one of the regulatory factors determining our body constitution and disease susceptibility, varies significantly, depending on the population and living environment. Recently, the integrated Human Microbiome Project (iHMP) explored the temporal dynamics of the microbiome and host by a multiomics approach and described the interrelationship between microbiota and diseases, including diabetes (Zhou et al., 2019) and inflammatory bowel disease (Lloyd-Price et al., 2019), and preterm birth (Fettweis et al., 2019).

In this study, we focused on the oral microbiome, the second largest and diverse microbiota harboring more than 700 species of bacteria (Kilian, 2018), and developed an oral metagenomics as one of the TMM multiomics analysis pipelines. The oral microbiota has been shown to be involved in the onset of various oral diseases, such as caries, periodontal disease, and cancer, by producing various noxious metabolites in the oral cavity (Willis and Gabaldón, 2020). Oral microbiomes seem to play critical roles in modulating the host response not only in the oral cavity but also in the whole body by acting as pathogens and/or changing metabolic pathways in the host (Leishman et al., 2012; Zheng et al., 2015). Accumulating lines of evidence support the notion that oral microbiota are closely related to systemic diseases (Graves et al., 2019; Willis and Gabaldón, 2020), including rheumatoid arthritis (Chen et al., 2018), adverse pregnancy outcomes (Cobb et al., 2017), and cardiovascular disease (Bryan et al., 2017). Therefore, comprehensive analysis of the microbiome together with the host genome and metabolome is a cutting-edge strategy for public health surveillance and disease risk prediction.

In this report, we introduce how we have conducted a baseline oral microbiome study in the ToMMo project. We also present the results of 16S ribosomal RNA (rRNA) gene analysis using saliva and plaque of 1,289 cohort participants, in which 834 were from the TMM CommCohort Study, and 455 were from the TMM BirThree Cohort Study. The former and the latter were exploited as discovery and validation cohorts, respectively. Our datasets revealed the differences in microbial composition and community structure between saliva and plaque and indicate the correlation between microbial species in saliva and the severity of periodontal disease.

## Materials and Methods

### Study Population, Design, and Samples

The cohort studies of the TMM project were approved by the ethics committees of Tohoku University and Iwate Medical University. The ToMMo baseline oral microbiome study was approved by the ethics committee of Tohoku University. All adult participants signed an informed consent form, and informed consent was obtained from a parent of minors.

We recruited participants from the baseline oral microbiome study at seven Community Support Centers in Miyagi Prefecture. On the day of the visit, participants completed an informed consent form, physiological measurements, oral examination and biospecimen collection. The details of all assessments, collections, and measurements were as previously reported (Kuriyama et al., 2016; Hozawa et al., 2020). We organized a team of 133 dentists who were given specific instructions regarding the oral examination methods in the ToMMo cohort studies. Details of oral examinations included dental and periodontal statuses, including third molar, occlusal, and oral hygiene statuses, conditions of soft tissue, frenal attachment, oral tori, and temporomandibular junction function.

A dental questionnaire was administered as reported (Tsuboi et al., 2020). Supragingival plaques were collected from right and left molar teeth [#16 and #26 under the Fédération Dentaire Internationale (FDI) Two-Digit system] by a sterile Gracey curette and suspended in 1 ml saline in a standardized tube. If the index first molar was missing, the sample was obtained from the second molar teeth instead. If both the first and second molars were absent, the sample was collected from the premolar teeth closest to the missing index teeth. Up to 2 ml of unstimulated whole mouth saliva was collected into a 50-ml sterile tube. A tongue swab specimen was collected from the surface of the tongue using a spoon-shaped plastic scraper, and then the scraper was dipped into 1 ml saline in a standard tube. Collection was conducted between 9 am and noon or 1 pm and 4 pm, and biospecimens were immediately frozen at -80°C.

Oral supragingival plaque, saliva and tongue swab biospecimens were transported on dry ice to the TMM biobank located at Tohoku University and immediately deidentified (Takai-Igarashi et al., 2017). Saliva was thawed once at 4°C and divided into half if there were more than 200 μl. One-half of the saliva was centrifuged at 3,000 × g for 10 min in a microcentrifuge tube and separated into the cell-free supernatant saliva and the cell containing sediment. Six types of oral biospecimens, *i.e.*, whole saliva without centrifugation, supernatant saliva, salivary sediment, supragingival plaque from right molar teeth, supragingival plaque from left molar teeth and tongue swab, were registered in the TMM biobank and stored at -80°C. LabVantage laboratory information management system (LIMS) (ver. 6 and ver. 8; LabVantage Solutions, Somerset, NJ) was used to manage the biospecimen information and to leave audit trails in the TMM biobank.

### Oral Microbiome Analysis

To analyze the saliva and plaque microbiota, we selected participants who met the following criteria: *i*) aged between 45 and 69 years, *ii*) had more than 20 teeth, and *iii*) underwent a dental examination and biospecimen collection by one dentist at the Sendai Community Support Center. In this study, we selected samples collected by one dentist. Therefore, unexpected variation of data derived from differences in minor maneuver seemed to be prevented. Of the 1,919 participants that met these three criteria, 1,349 participants were randomly selected for the microbiome analysis. There were 1,349 and 1,345 analyzed samples for supragingival plaque from right molar teeth and whole saliva, respectively. In this study, we defined four severity categories of periodontal disease by the percentage of teeth with periodontal pocket depth (PPD) ≥ 4 mm: healthy (0%), mild (> 0% and ≤ 20%), moderate (> 20% and ≤ 50%), and severe (> 50%).

### Amplification and Sequencing

The composition and diversity of the oral microbiome were assessed by high-throughput sequencing of 16S rRNA gene amplicons by using the Illumina MiSeq Platform (Illumina, Inc., San Diego, CA). The plaque and saliva samples from 1,349 participants were subjected to DNA extraction using the PowerSoil DNA Isolation Kit (MO BIO, Carlsbad, CA) according to the manufacturer’s protocol. Sequencing libraries were prepared using a two-step PCR method for targeting the V4 region (typically 258 or 259 bp) of the 16S rRNA gene as previously described (Sato et al., 2015). In brief, the first PCR was conducted using gene-specific primers, followed by the second PCR using separate indexing primers that fused Illumina sequencing adaptors plus dual barcodes to the sample amplicons. Ex Taq DNA Polymerase (TaKaRa Bio Inc., Japan) was used for PCR amplification. The pooled library was then quantified using the Qubit 2.0 Fluorometer and dsDNA HS Assay Kit (Life Technologies, Carlsbad, CA) and diluted to a final concentration of 12 pM with 50% PhiX. Sequencing was performed using the MiSeq Reagent Kit v3 (Illumina, Inc.) by a 300 bp paired-end sequencing protocol according to the manufacturer’s instructions, and 28.15 million paired-end reads were obtained in total. The samples had a mean read pair count of 20,934 and 22,228 and a maximum read pair count of 66,325 and 76,399 in saliva and plaque, respectively.

### Amplicon Sequence Variants

Sequence data for the 16S rRNA gene amplicons were analyzed using the QIIME2 platform, version 2018.11 (Bolyen et al., 2019). For all paired reads, the first 20 bases of both sequences were trimmed to remove primer sequences, the bases after position 200 were truncated to remove low-quality sequence data, and potential amplicon sequencing errors were corrected using DADA2 (Callahan et al., 2016) to produce an amplicon sequence variant (ASV) dataset. The resultant ASVs were aligned using MAFFT (Katoh and Standley, 2013) and then used to construct a phylogenetic tree with FastTree2 (Price et al., 2010).

The α- and β-diversity metrics were estimated from a subsampled ASV dataset, with 10,000 sequences per sample. Each ASV was identified using a Naïve Bayes classifier trained on 16S rRNA gene sequences from the Greengenes dataset (release 13_8) (Bokulich et al., 2018). All reads were assigned to total 232 and 259 ASVs at the species level in saliva and plaque, respectively.

Principal coordinate analysis (PCoA) and other statistical analyses were performed using custom R and Python scripts. ASVs detected one or more times in less than 500 samples were defined as minor. The dataset of 1,289 participants who had over 10,000 high-quality reads per person in the saliva and plaque, excluding withdrawal of the consent before February 3, 2020, have been released in the jMorp database (https://jmorp.megabank.tohoku.ac.jp/) and the Integrated Database of Tohoku Medical Megabank (dbTMM; http://www.dist.megabank.tohoku.ac.jp/index.html).

### Statistics

All statistical analyses were performed using R 3.5.3 (https://www.r-project.org/) or SAS 9.4 software (SAS Institute Inc., Cary, NC). Probability values *p* < 0.05 were considered to indicate statistical significance. Co-occurrence network analysis was performed based on the relative abundance of ASVs using Cytoscape 3.7.2 software (http://www.cytoscape.org/). Nodes and edges were filtered by correlation value (Spearman’s correlation coefficient ≥ 0.4 or ≤ -0.4, *p* < 0.05) and visualized.

## Results

### Study Design and Sample Collection

The ToMMo baseline oral microbiome study was performed on the participants of the TMM cohort studies. During the first half of the baseline survey (October 2013–May 2016), this study targeted more than 25,000 participants who received a dental checkup either in the TMM CommCohort Study or in the TMM BirThree Cohort Study. The oral cavity has various microbial habitats, such as teeth, buccal mucosa, soft and hard palates, and tongue, each of which forms a species-rich unique microbiota (Kilian, 2018).

In the ToMMo baseline oral microbiome study, we recruited participants at seven Community Support Centers (Kesennuma, Osaki, Ishinomaki, Tagajo, Sendai, Iwanuma, and Shiroishi) in Miyagi Prefecture, which were established for the voluntary recruitment and health assessment of participants. On the day of the visit, participants completed an informed consent form and physiological measurements, oral examination and biospecimen collection were performed as reported (Kuriyama et al., 2016; Hozawa et al., 2020). A comprehensive whole mouth oral examination was performed by trained dentists, and details of oral examinations and dental questionnaires were as previously reported (Tsuboi et al., 2020).

We aimed to obtain three types of oral biospecimens, *i.e.*, saliva, dental plaque, and tongue swab, from participants. Saliva contains microbiota exfoliated from various habitats of the oral cavity and is a suitable sample for evaluating the entire oral microbiome. Dental plaque exists close to the foci of caries and periodontal diseases, two major diseases of the oral cavity, and is deeply involved in the development and progression of these diseases. The tongue is the largest microbial habitat in the oral cavity, and various kinds of microbial species, both aerobic or anaerobic, inhabit the tongue coating. Therefore, we collected supragingival plaque from right and left molar teeth, unstimulated whole mouth saliva, and tongue swab specimens from a total of 25,101 participants in this study (**Table 1**). These collections were conducted by trained dentists, and biospecimens were immediately frozen at -80°C. These oral biospecimens were transported to the TMM biobank and immediately deidentified (Takai-Igarashi et al., 2017).

**Table 1:** Overview of the oral biospecimens in the ToMMo baseline oral microbiome study.

		Number of times to visit to the Community Support Centers			
		1st visit	2nd visit	3rd visit	Total
Participants with oral biospecimens		25,014	86	1	25,101
Saliva	Whole	24,868	85	1	24,954
	Supernatant	24,423	83	0	24,506
	Sediment	24,464	84	0	24,548
Supragingival plaque	Right molar tooth	24,889	82	1	24,972
	Left molar tooth	24,876	82	1	24,959
Tongue swab		24,988	82	1	25,071

**Figure 1:**
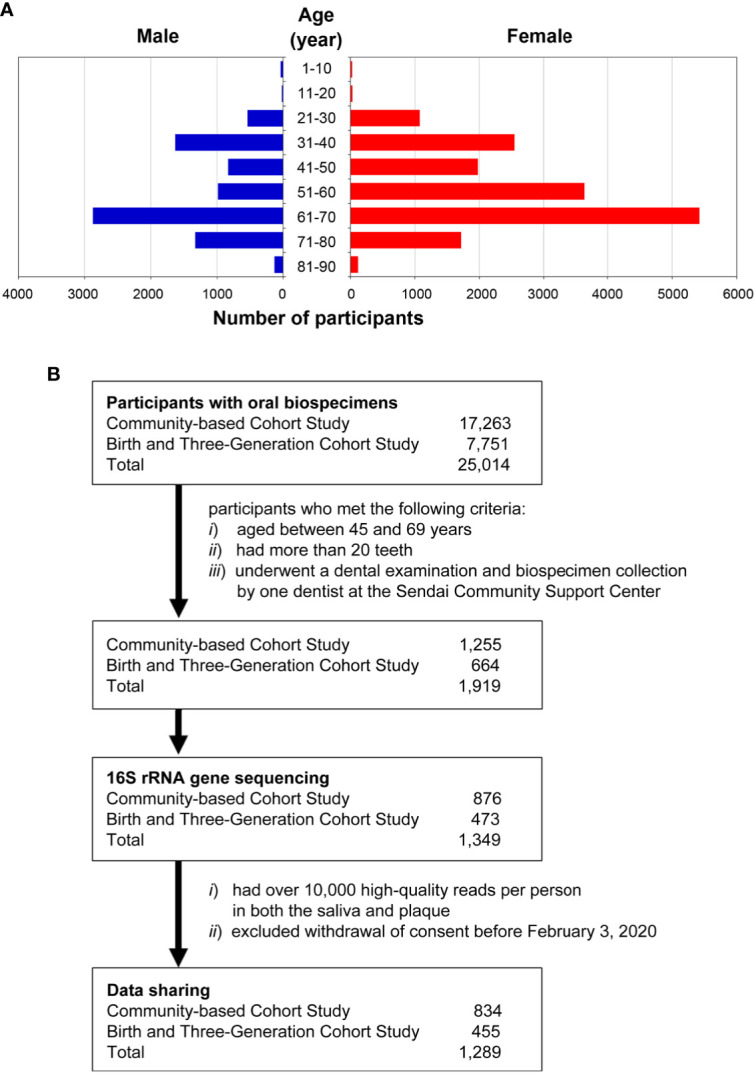
Overview of the participants in the ToMMo baseline oral microbiome study. **(A)** Sex and age distribution of the participants (n = 24,913) in the ToMMo baseline oral microbiome study. Of the 25,014 individuals from which oral biospecimens were collected, 101 were excluded due to missing information regarding gender, date of birth, or dental examination. **(B)** Number of participants registered in the TMM CommCohort Study and in the TMM BirThree Cohort Study. The flow chart shows the criteria for selecting the participants whose data were analyzed by 16S rRNA gene sequencing.

The age distribution of the participants is shown in **Figure 1A**. The average age was 54.9 ± 15.1 years, with a range from 5 to 90 years, and the group of participants aged 61–70 years was the largest, accounting for 33.3% of the participants. There were 17,263 and 7,751 participants in the TMM CommCohort Study and in the TMM BirThree Cohort Study, respectively (**Figure 1B**).

Several studies have reported that the human microbiota varies greatly by geographical location and/or ethnicity (Gupta et al., 2017). Therefore, constructing a Japanese reference database covering the microbiota inhabiting multiple sites in the oral cavity is important for research on diseases affected by oral microbiota in the Japanese population. In particular, this database is essential for studying oral diseases. To analyze the saliva and plaque microbiotas, we randomly selected 1,349 participants from 1,919 participants who met the following criteria: *i*) between 45 and 69 years of age, *ii*) more than 20 teeth and *iii*) underwent a dental examination and biospecimen collection by one dentist at the Sendai Community Support Center.

There were 1,349 and 1,345 analyzed samples for supragingival plaque from right molar teeth and whole saliva, respectively. We designed two-phase discovery and validation analyses to identify microbial taxa of interest for oral disease states using two closely related but independent cohorts: the TMM CommCohort Study as the discovery cohort and the TMM BirThree Cohort Study as the validation cohort. Of the 1,349 analyzed participants, 876 participants were from the TMM CommCohort Study, and 473 participants were from the TMM BirThree Cohort Study (**Figure 1B**).

### Species Diversity (α-Diversity) Correlates With Periodontal Disease Severity in Oral Microbiota

**Figure 2:**
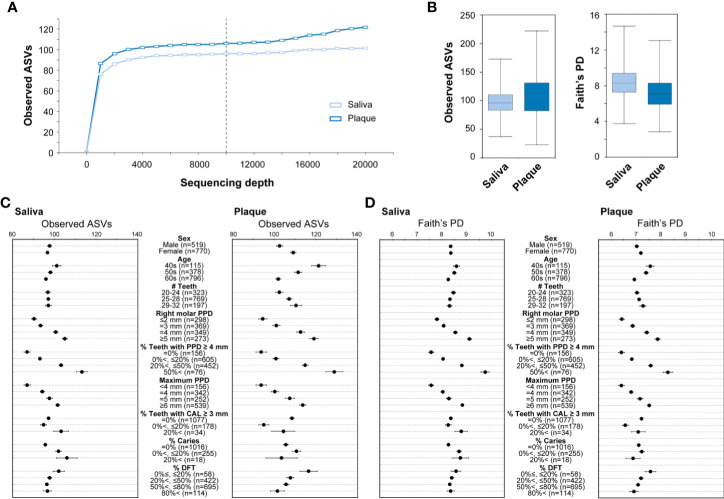
The α-diversity of the microbiota in saliva and plaque. **(A)** Rarefaction curves based on the observed ASVs. The colors indicate sample type: saliva (light blue) and plaque (blue). The vertical black dashed line indicates the 10,000-read rarefaction threshold. **(B)** The α-diversity in saliva and plaque. The box plots show two different indices: the number of observed ASVs (left) and Faith’s phylogenetic diversity (right). Data obtained from 1,289 participants in the TMM CommCohort Study and the TMM BirThree Cohort Study were analyzed and used for discovery and validation, respectively. **(C, D)** Number of observed ASVs **(C)** and Faith’s phylogenetic diversity **(D)** compared under the indicated conditions. The dots indicate the means; the error bars indicate the standard errors (SEs). PD, phylogenetic diversity; PPD, periodontal pocket depth; CAL, clinical attachment level; DFT, decayed and filled teeth.

With the aim of building a database of the human microbiome in the TMM project, we established a new pipeline for microbiome 16S rRNA gene analysis by improving the methods used in previous oral microbiome studies (Sato et al., 2015; Yamagishi et al., 2016). As described in the previous section, we analyzed the microbiota of saliva and plaque obtained from 1,349 TMM cohort participants as the first trial to compare their profiles. To determine whether all the ASVs present in the dataset were recovered in the 16S amplicon sequencing, we carried out rarefaction analysis (**Figure 2A** and **Supplementary Figure S1**). After quality checks and subsampling, we obtained 10,000 high-quality reads in both the saliva and plaque samples of the 1,289 participants (519 males and 770 females, mean age 60.16 ± 6.39 years). The clinical characteristics of 834 participants from the TMM CommCohort Study (discovery cohort) and 455 participants from the TMM BirThree Cohort Study (validation cohort) are described in **Tables 2** and **3**, respectively.

**Table 2:** Dental characteristics of the study population in the TMM CommCohort Study.

Periodontal Disease Severity	Healthy	Mild	Moderate	Severe
# Participants	110	399	287	38
Age (Years)				
Mean ± S.D.	53.3 ± 7.1	60.1 ± 6.8	59.9 ± 6.6	59.9 ± 6.1
Sex				
Female	72	262	179	22
Male	38	137	108	16
# Teeth				
Mean ± S.D.	26.3 ± 2.7	26.3 ± 2.5	26.1 ± 2.7	25.1 ± 3.1
# Teeth with PPD ≥ 4 mm				
Mean ± S.D.	0	2.7 ± 1.4	7.8 ± 2.1	16.0 ± 3.4
# DFT				
Mean ± S.D.	14.1 ± 5.2	14.4 ± 4.9	14.5 ± 4.6	12.7 ± 5.1

S.D., standard deviation; PPD, periodontal pocket depth; DFT, decayed and filled teeth.

**Table 3:** Dental characteristics of the study population in the TMM BirThree Cohort Study.

Periodontal Disease Severity	Healthy	Mild	Moderate	Severe
# Participants	46	206	165	38
Age (Years)				
Mean ± S.D.	59.4 ± 5.9	61.1 ± 5.5	60.4 ± 5.7	60.2 ± 6.2
Sex				
Female	30	101	90	14
Male	16	105	75	24
# Teeth				
Mean ± S.D.	26.4 ± 2.6	26.4 ± 2.7	25.9 ± 2.8	25.7 ± 2.9
# Teeth with PPD ≥ 4 mm				
Mean ± S.D.	0	2.7 ± 1.6	8.5 ± 2.2	16.5 ± 3.7
# DFT				
Mean ± S.D.	13.7 ± 4.8	13.8 ± 5.0	14.0 ± 5.3	12.7 ± 6.4

S.D., standard deviation; PPD, periodontal pocket depth; DFT, decayed and filled teeth.

We analyzed the species richness, which is often referred to as α-diversity, in the oral microbial communities by comparing the number of ASVs among the individuals. The α-diversity was calculated using combined data from the TMM CommCohort Study and the TMM BirThree Cohort Study. Each 10,000 high-quality reads from the saliva and plaque were employed to assign ASVs. There was considerably more intra-member variability in the ASV numbers in the plaque samples than in the saliva samples (**Figure 2B**). The influence of the ratio of teeth with deep pockets (PPD ≥ 4 mm), PPD of right molar and maximum PPD on the number of ASVs was statistically significant in both saliva and plaque samples, suggesting that the richness of oral bacterial communities was relevant to dental characteristics (**Figure 2C**).

To calculate the α-diversity, we employed Faith’s phylogenetic diversity, which is one of the indices for α-diversity (Faith, 1992). We confirmed that the α-diversity was increased stepwise and that the increase was markedly correlated with the ratio of teeth with deep pockets in both saliva and plaque samples (**Figure 2D**). Our data also indicated that the phylogenetic diversity values were higher in younger individuals. The results were consistent with the results calculated by the Shannon diversity index and Pielou’s evenness index for α-diversity metrics (**Supplementary Figure S2**). These results demonstrate that the increase in species diversity is a distinctive feature of oral microbiota accompanying periodontitis. This conclusion is consistent with that reported by Curtis et al., 2020.

According to the α-diversity analysis results, we decided to focus on the association of periodontal disease severity with microbial communities in the saliva and plaque. In this study, we defined the severity of periodontal disease based on the ratio of teeth with deep pockets (PPD ≥ 4 mm) as four classes: healthy (0%), mild (20% or less), moderate (higher than 20% and 50% or less), and severe (higher than 50%).

### Significant Differences in Salivary and Plaque Microbiome Structures (β-Diversity)

**Figure 3:**
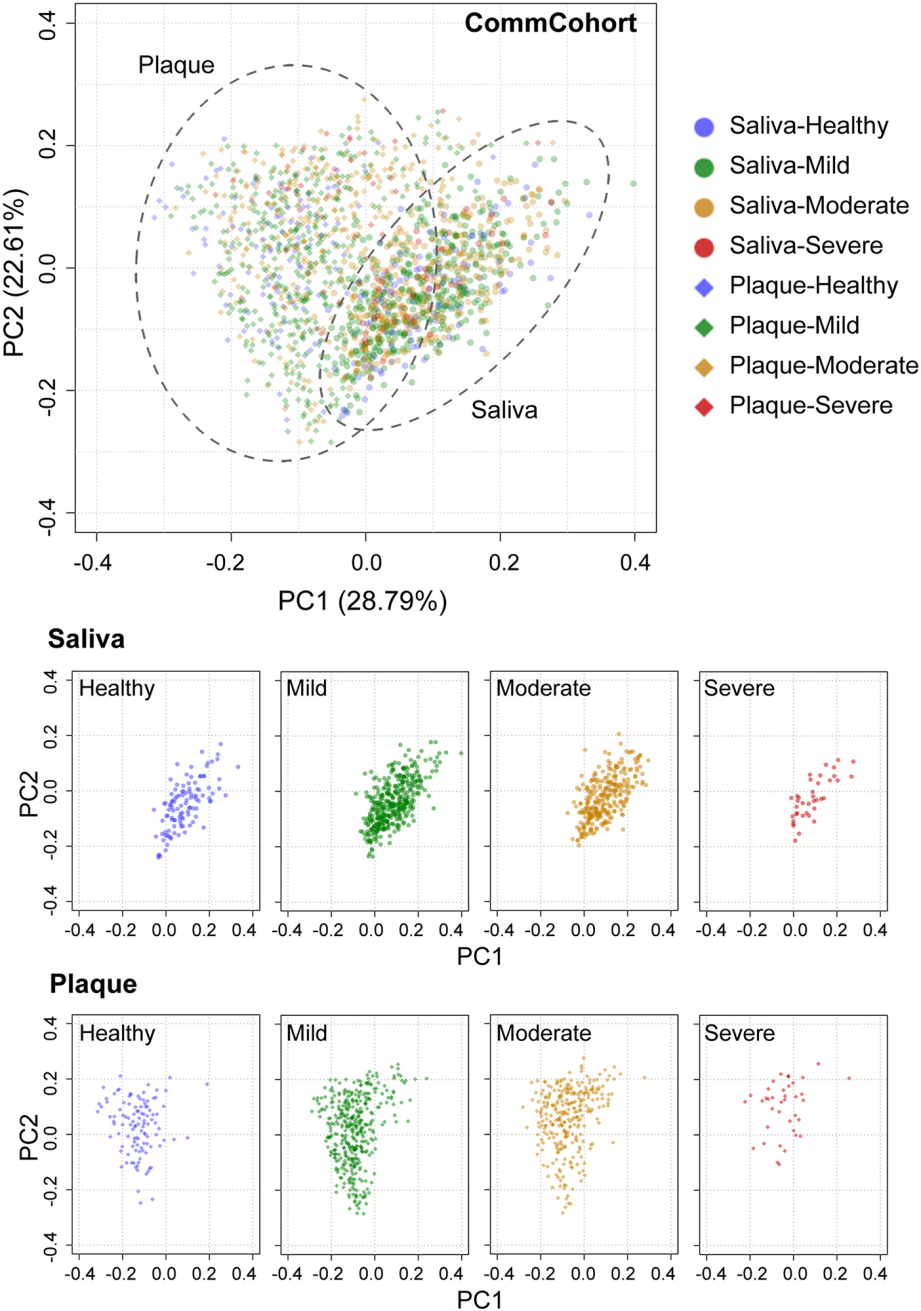
PCoA plots of oral microbial communities in the TMM CommCohort Study based on weighted UniFrac distance. Each dot represents the microbial community in the saliva (circles, n = 834) and plaque (diamonds, n = 834) of one individual. The dots in the top panel were separated into eight groups by sample type, which are shown at the bottom. The severity of periodontal disease was classified into four categories based on the ratio of teeth with deep pockets: healthy (0%, blue), mild (> 0% and ≤ 20%, green), moderate (> 20% and ≤ 50%, yellow), and severe (> 50%, red).

We then analyzed the phylogenetic diversity among the oral microbiota structures, which is often referred to as β-diversity, in participants of the TMM CommCohort Study (discovery cohort; n = 834) using PCoA. We conducted PCoA by categorizing the discovery cohort participants into four severity categories (*i.e.*, healthy, mild, moderate, and severe) of periodontal disease depending on the scores shown in **Figure 2C**. As shown in **Figure 3**, the PCoA plot based on the weighted UniFrac distance displayed apparent differences between the saliva (middle panels) and plaque (lower panels) samples, while variations in the PCoA plot among the four severity categories were not found. We also analyzed the β-diversity of saliva and plaque microbiomes in participants of the TMM BirThree Cohort Study (validation cohort; n = 455) independently and found that the findings discovered in the TMM CommCohort Study were reproducible in the TMM BirThree Cohort Study (**Supplementary Figure S3**). These results indicate that the microbial community compositions differed notably between saliva and plaque samples, regardless of periodontal disease status.

### Relative Microbial Abundance Differs in Saliva and Plaque

**Figure 4:**
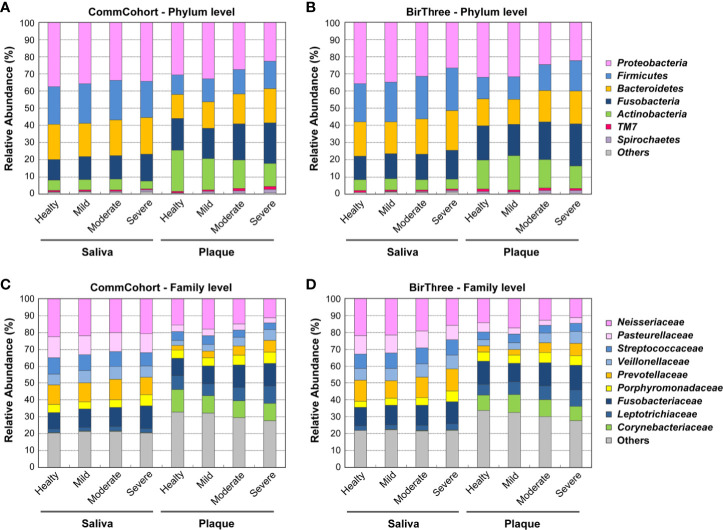
Comparison of microbial composition in saliva and plaque. **(A, B)** Stacked bar plots showing the relative abundance of microbial taxa at the phylum level in the TMM CommCohort Study **(A)** and the TMM BirThree Cohort Study **(B)**. Data from these cohorts were used for discovery and validation, respectively. The labels indicate the phyla with mean relative abundances ≥ 2% in at least one group. The remaining phyla were binned together. **(C, D)** Stacked bar plots showing the mean relative abundances of microbial taxa at the family level in the TMM CommCohort Study **(C)** and the TMM BirThree Cohort Study **(D)**. The labels indicate the top 9 taxa at the family level, and the remaining families were binned together.

Comparison of relative microbial abundance at the phylum level indicates that the abundance ratios of the phylum *Firmicutes* (**Figure 4A**, blue) and the phylum *Actinobacteria* (green) were higher and lower, respectively, in saliva than in plaque, regardless of oral disease condition. This observation in saliva and plaque microbiomes was reproducible in participants of the TMM BirThree Cohort Study (**Figure 4B**).

Similarly, when we examined the relative microbial abundance at the family level, we found that the families *Neisseriaceae* (**Figure 4C**, pink) and *Fusobacteriaceae* (dark navy), both of which are known as resident bacteria in the oral cavity, were dominant in both saliva and plaque samples. The former was more abundant in saliva than in plaque, while the latter was more abundant in plaque than in saliva. Similarly, the relative abundances of the family *Prevotellaceae* (orange) and the family *Veillonellaceae* (light blue) were higher in saliva than in plaque. In addition, these two families in plaque samples showed a tendency to increase in participants with periodontal disease. Our results are consistent with the finding that periodontal disease was found frequently in a group with a higher ratio of the *Prevotellaceae* and *Veillonellaceae* families (Takeshita et al., 2009).

The dataset also showed that the relative abundances of the families *Leptotrichiaceae* (navy) and *Corynebacteriaceae* (green) were higher in plaque than in saliva. It has been reported that *Corynebacterium matruchotii* comprises the central filament of corn-cob formations in mature dental plaque and is involved in tartar formation (Paster et al., 2001). We also analyzed the β-diversity of saliva and plaque microbiomes in participants of the validation cohort, and these findings in the discovery cohort were substantially validated in the validation cohort (**Figure 4D**).

### Changes in Microbial Abundance According to Periodontal Disease Severity

**Figure 5:**
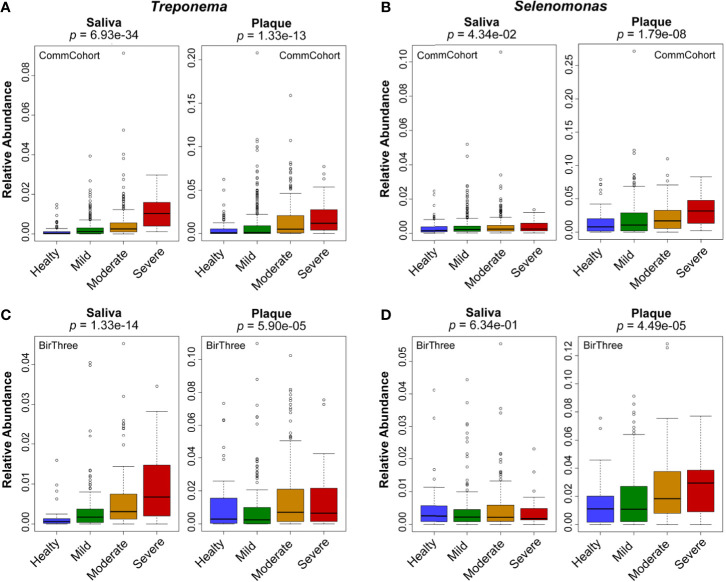
Changes in relative microbial abundance at the genus level according to periodontal disease severity. Box plots showing the relative abundance of the genera *Treponema* **(A, C)** and *Selenomonas* **(B, D)** in saliva and plaque among the four severity categories of periodontal disease. Data from two independent cohorts, the TMM CommCohort Study **(A, B)** and the TMM BirThree Cohort Study **(C, D)**, were used for discovery and validation, respectively. The significance of the differences was estimated by using logistic regression analysis.

To address details of the dysbiotic nature of oral microbiota in periodontal disease severity, we then searched bacterial genera whose relative abundance was featured in one of the four categories of periodontal disease in either saliva or plaque samples. In the discovery cohort (the TMM CommCohort Study), a significant increase in the genera *Treponema* and *Selenomonas* was identified in our dataset (**Figures 5A, B**), and both of these genera are well known as periodontal disease-causing bacteria. The genus *Treponema* was significantly increased in saliva of the severe periodontal disease category and in plaque (**Figure 5A**). Of note, the genus *Treponema* contains *Treponema denticola*, which has been implicated as a key pathogen in periodontal disease (Holt and Ebersole, 2005). In contrast, the genus *Selenomonas* was significantly increased in plaque of the severe periodontal disease category (**Figure 5B**). In addition, the genera *Tannerella* and *Prevotella* also showed significant differences in relative abundance among the four severity categories in saliva and plaque, respectively (**Supplementary Figure S4**).

These results clearly demonstrate that relative microbiome abundance changes according to the severity of periodontal disease. Substantial changes can be observed at the family and genus levels. Of note, in certain cases, the directions of these changes are distinct in saliva and plaque; most significantly, at the genus level, the increase occurs preferentially in saliva (*Treponema*) or in plaque (*Selenomonas*). The results were reproducible in the microbiome data of the validation cohort (TMM BirThree Cohort Study) (**Figures 5C, D**).

### PCoA Plots of the Oral Microbial Community Based on Unweighted UniFrac Distance

**Figure 6:**
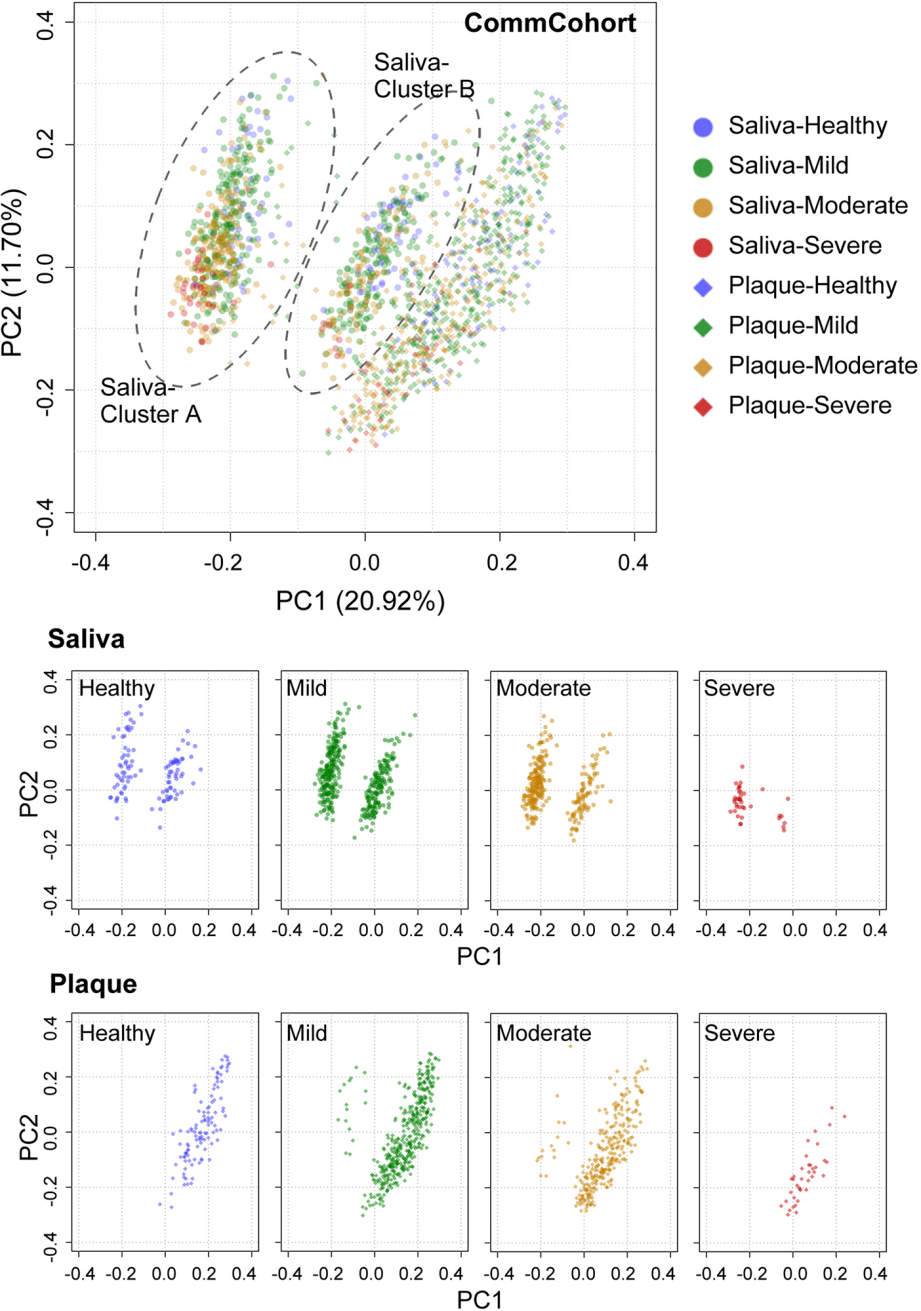
PCoA plots of oral microbial communities in the TMM CommCohort Study based on unweighted UniFrac distance. Each dot represents the microbial community in the saliva (circles, n = 834) and plaque (diamonds, n = 834) of one individual. The dots in the top panel were separated into eight groups by sample type, which are shown at the bottom. A distinct separation of the saliva samples that fall into two clusters (cluster A and cluster B) according to the PC1 axis is shown. The severity of periodontal disease was classified into four categories based on the ratio of teeth with deep pockets: healthy (0%, blue), mild (> 0% and ≤ 20%, green), moderate (> 20% and ≤ 50%, yellow), and severe (> 50%, red).

The PCoA plot based on unweighted UniFrac distance, which emphasizes the abundance change in minor lineages, showed differences in the microbial community structure by sample type in the discovery cohort (TMM CommCohort Study) (**Figure 6**). The results show very good agreement with those based on the weighted UniFrac distance (**Figure 3**), but it should be noted that PCoA based on unweighted UniFrac distance revealed a distinct separation of the saliva samples that fall into two clusters according to the PC1 axis (**Figure 6**). We referred to the left and right clusters in saliva as clusters A and B, respectively. In contrast, only one component appeared in the PC1 axis of plaque samples. Similar results were obtained in saliva and plaque microbiotas in the validation cohort (TMM BirThree Cohort Study) (**Supplementary Figure S5**). Thus, the PCoA plot based on the unweighted UniFrac distance revealed abundance differences in minor lineages.

### Association Between Severe Periodontal Disease and Minor Taxa in Saliva

**Figure 7:**
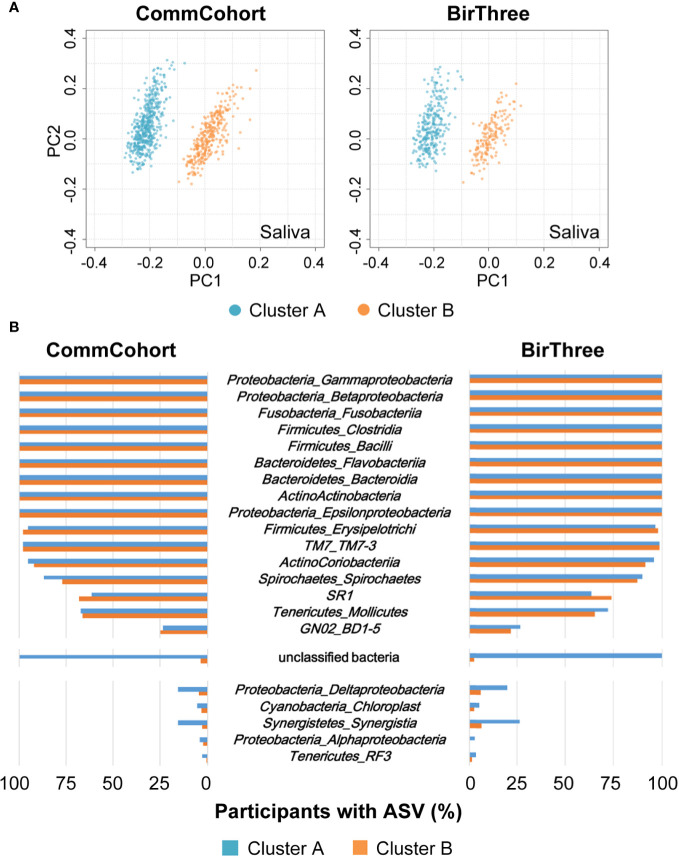
Association between salivary microbiome clusters and minor taxa. **(A)** PCoA plots of the saliva microbial communities based on unweighted UniFrac distances in the TMM CommCohort Study (left) and the TMM BirThree Cohort Study (right). Two distinct clusters of the microbial community, one on the left (cluster A, light blue) and another on the right (cluster B, orange), are shown. **(B)** The bar graph shows the frequency at which the indicated microbial taxa were detected at the class level in the saliva samples from participants of the TMM CommCohort Study (left) and the TMM BirThree Cohort Study (right). Note that several unclassified bacteria were found in the participants in cluster A.

When we filtered out the low frequency ASVs detected once or more in less than 500 samples to exclude minor taxa, PCoA dots concentrated on one cluster in saliva samples of the discovery and validation cohorts (**Supplementary Figures S6A** and **S6B**). However, as the PCoA plot based on the unweighted UniFrac distance revealed the abundance changes in minor lineages, we decided to examine whether there are unclassified bacteria in clusters A and B (**Figure 7**). While we found that the distribution of major bacterial classes was almost comparable in clusters A and B (**Supplementary Figure S7**), unclassified bacteria were detected almost exclusively in cluster A in saliva samples of nearly all participants. Unclassified bacteria were rarely found in cluster B. Thus, the existence of susceptible minor ASVs appears to contribute to the creation of an unusual cluster.

**Figure 8:**
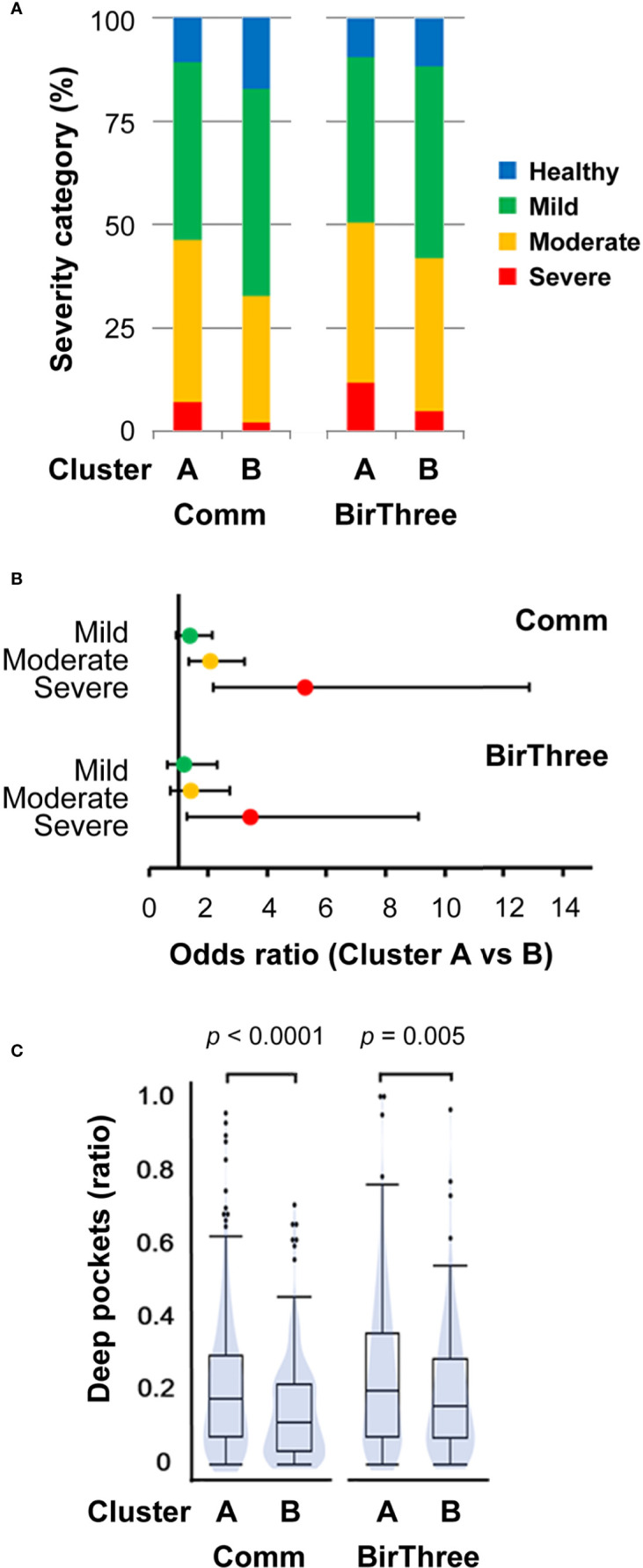
Association between salivary microbiome cluster and periodontal disease severity. **(A)** Distribution of the four periodontal disease severity categories among participants in clusters A and B. **(B)** The odds ratios adjusted for age and the number of teeth with periodontal disease falling into each severity category (with 95% confidence intervals) for participants in cluster B compared to those in cluster A. **(C)** Violin plots of the relative number of deep pockets (≥ 4 mm). The significance of the differences was estimated by using unpaired Student’s t-tests. Comm, TMM CommCohort Study; BirThree, TMM BirThree Cohort Study.

Since there are two clusters in the unweighted UniFrac distance matrix, we next focused on the differences in the two clusters of the saliva microbiome. Therefore, we evaluated the dental condition of participants in each cluster and found that the percentages of participants belonging to the severe periodontal disease categories were higher in cluster A (7.02% and 11.72%, respectively) than in cluster B (2.18% and 4.85%, respectively) both in the discovery cohort (TMM CommCohort Study) and the validation cohort (TMM BirThree Cohort Study) (**Figure 8A**). In the discovery cohort (TMM CommCohort Study), the odds ratio for the risk of having severe periodontal disease was adjusted as 5.26 (95% confidence interval (Cl) 2.15 to 12.86; *p* = 0.0003) in participants in cluster B compared to those in cluster A (**Figure 8B**). Similarly, the relative number of deep pockets was significantly higher in cluster A (0.18, median) than in cluster B (0.12, median) (**Figure 8C**). These results were consistent in the validation cohort (TMM BirThree Cohort Study) (**Figures 8B, C**).

These results revealed that unknown bacteria are more enriched in cluster A than in cluster B in saliva and that participants with severe periodontal disease are more enriched in cluster A than in cluster B. Taken together, these results suggest the contribution of certain unknown bacteria in the unclassified fraction to the severity of periodontal disease, supporting the notion that novel microbes associated with oral diseases may reside in the unclassified fraction.

### Microbiome Network and Community Structure

**Figure 9:**
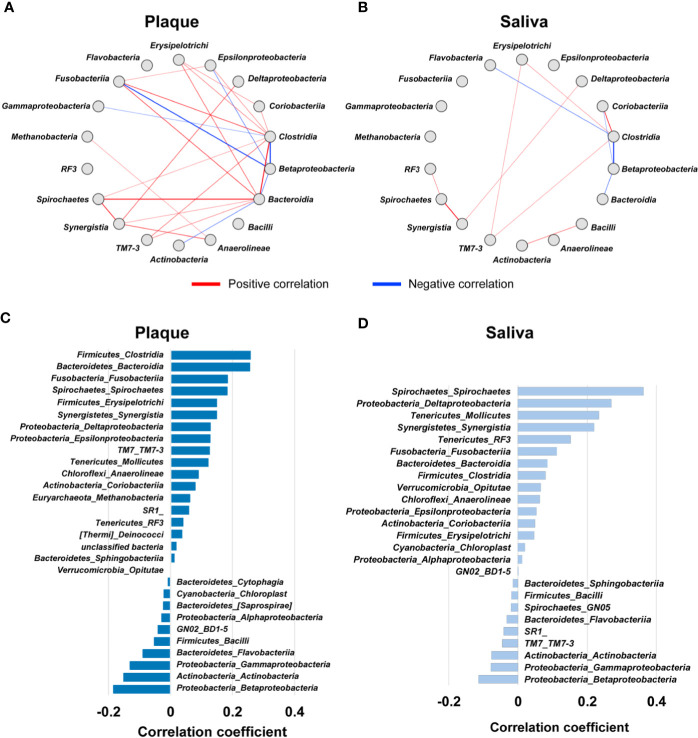
Co-occurrence network of oral microbial communities. **(A, B)** Significant correlations of microbial taxa (Spearman’s correlation coefficient ≥ 0.4 or ≤ -0.4, *p* < 0.05) in plaque **(A)** and saliva **(B)**. Each node represents a microbial taxon at the class level. The direction and strength of the correlations between two taxa is represented by the thickness of the red (positive) or blue (negative) lines. **(C, D)** Bar plot indicating Pearson’s correlation coefficients between the ratio of deep pockets (≥ 4 mm) and the relative microbial abundance in plaque **(C)** and saliva **(D)**. The microbial taxa at the class level with *p* ≤ 0.05 are arranged in descending order by correlation coefficient.

To examine the oral microbial ecology in saliva and plaque, we performed co-occurrence network analyses at the class level based on the relative abundance of ASVs. As shown in **Figures 9A, B**, we identified 18 microbial taxa that have correlative relationships in either or both saliva and plaque. In this figure, the direction and strength of correlations between two taxa are shown by the thickness of red (positive) or blue (negative) lines. The correlative relationships of the plaque microbiome are stronger than those of salivary microbiome.

In plaque, six microbial taxa, *i.e.*, the classes *Bacteroidia*, *Clostridia*, *Erysipelotrichi, Fusobacteriia*, *Spirochaetes*, and *Synergistia* were found to positively correlate with each other (**Figure 9A**). In particular, the classes *Bacteroidia* and *Spirochaetes*, showed the strongest positive correlation. The class *Bacteroidia* contains *Porphyromonas gingivalis* and *Tannerella forsythia*, which are very strong periodontal pathogen species together with *Treponema denticola* in the class *Spirochaetes* (Holt and Ebersole, 2005). When we analyzed the correlation of the relative abundance of the microbiome with the relative number of deep pockets, we found that these 6 microbial taxa were listed in the top 6 with a positive correlation (**Figure 9C**), indicating that these taxa proliferate concomitantly in periodontal disease conditions. In contrast, there were only weak correlations among microbial taxa in saliva (**Figure 9B**). We surmise that this is probably due to the composited microbiome structure derived from various oral surfaces.

The class *Synergistia* had a positive correlation with *Spirochaetes* in saliva and plaque and *Bacteroidia* in plaque. *Synergistia* is known to be anaerobic gram-negative bacteria that resides in sites of human disease, such as periodontal disease (Horz et al., 2006). It has been reported that *Synergistia* contains the species *Fretibacterium fastidiosum* and *Fretibacterium* sp. in human oral taxon 360, and both species have been isolated as common periodontitis-associated species by 16S rRNA gene sequencing in microbiome studies (Curtis et al., 2020). Indeed, correlation analysis of the relative abundance of *Synergistia* and the relative number of deep periodontal pockets showed a high correlation both in saliva and plaque (**Figures 9C, D**). The relative abundance of the other 7 taxa (*Deltaproteobacteria, Epsilonproteobacteria, TM7-3, Anaerolineae*, *Coriobacteriia*, *Methanobacteria*, and *RF3*) also showed a positive correlation with the relative number of deep pockets in plaque (**Figure 9C**), indicating that microbial species that were currently not identified as periodontal pathogens may also be present in these taxa. Current data showed that *Desulfomicrobium orale* in the class *Deltaproteobacteria* had a positive correlation with the ratio of deep pockets in plaque (**Supplementary Figure S8**).

The class *Betaproteobacteria* exhibited a strong negative correlation with *Clostridia* in plaque and saliva and *Fusobacteriia* in plaque. In plaque, *Actinobacteria* and *Betaproteobacteria* showed a moderate negative correlation with *Bacteroidia* (**Figure 9A**). Stepwise plaque formation has been shown to be achieved by sequential colonization from early colonizers to late colonizers and red complex bacteria species, which consist of keystone periodontal pathogens involved in the onset of periodontal diseases (Socransky et al., 1998; Kolenbrander et al., 2010; Kirst et al., 2015). The relative abundance of the classes *Betaproteobacteria* and *Actinobacteria* showed a negative correlation with the relative number of deep pockets in plaque (**Figure 9C**), supporting the notion that this sort of taxa balance is associated with the progression of periodontal diseases.

## Discussion

As the oral cavity is the initial entry point of the gastrointestinal tract, the oral microbiome may be involved in the pathogenesis or progression of systemic diseases. In this study, we approached the oral microbiome study by using independent discovery (TMM CommCohort Study) and validation (TMM BirThree Cohort Study) cohorts and selected microbial taxa of interest for oral disease. We found through 16S rRNA gene sequencing analysis of 1,289 participants, in which 834 participants in the discovery cohort and 455 participants in the validation cohort, significant differences in the microbial composition and community structure between saliva and plaque. We also found that the species diversities in both saliva and plaque were increased in correlation with the severity of periodontal disease. By means of co-occurrence network analysis, a strong association of microbial taxa in plaque with periodontitis-associated biofilm formation was detected. These results thus revealed unique features of the oral microbiome in plaque and saliva, which were associated with the severity of periodontal diseases.

As a biobank, the TMM biobank wishes to share data from various genome studies with the research community to advance diverse scientific fields. The microbiome dataset of the ToMMo baseline oral microbiome study has been released to public through the dbTMM and jMorp databases. The latter contains and has already been sharing large-scale cohort data in the TMM project, covering genomics, transcriptomics, and metabolomics along with clinical information. The integrative approach by using microbiome and multiomics is expected to serve for the discovery of novel targets for the treatment and prevention of oral diseases.

In this study, we analyzed the microbiota of whole saliva and supragingival plaque from adult TMM cohort participants by means of 16S rRNA gene amplicon sequencing and measured the composition and relative abundance of the oral microbial population. Showing very good agreement with a previous report by the Human Microbiome Project (HMP; Segata et al., 2012), the frequency of the phylum *Firmicutes* in plaque is reduced compared with that in saliva, while the relative abundance of the phylum *Actinobacteria*, especially the genus *Corynebacterium*, is increased in plaque compared with that in saliva. The relative abundances of the phyla *Proteobacteria* and *TM7* tended to be lower in the TMM cohort than in the HMP cohort in both saliva and plaque. This may be due to variations caused by differences in the experimental protocol, at least in part. Accumulating lines of evidence imply that the comparison of microbiome datasets from different research programs requires careful attention for variations. In this regard, large-scale microbiome analyses performed by a single facility would further improve the usability of genome/omics data supplemented with medical information in understanding human health.

Since saliva and supragingival plaque collection are less invasive, oral microbial studies employing saliva and plaque are commonly carried out. However, the relationship between salivary and plaque microbiota remains elusive. In this study, we clarified that the microbial ecology in saliva is different from that in plaque through co-occurrence network analysis of salivary and plaque microbiomes. The symbiotic relationship of the plaque microbiome is stronger than that of salivary microbiome, and the plaque microbiome at the class level has a strong correlation with periodontitis conditions. We plan to conduct a whole genome metagenomics analysis, which will provide sufficient taxonomic resolution to perform species-level association study with oral microbiome and oral diseases. Of note, in addition to the positive correlations of pathogenic microorganisms in plaque, we also identified negative correlations of the classes *Betaproteobacteria* and *Actinobacteria* with pathogenic microbial taxa in plaque. This result evokes an intriguing issue, *i.e.*, the microbiome switches from beneficial bacteria to pathogenic bacteria. However, considering the finding that the absolute amount of total oral bacteria is increased in the presence of periodontitis (Abusleme et al., 2013), quantitative analysis of the microbiome seems to be a prerequisite to fully understand the positive-negative relationships between oral microbiomes in disease conditions.

In addition, the mutual relationship in the salivary microbiome was much weaker than that in the plaque microbiome. Nonetheless, intriguingly, a positive correlation between the classes *Actinobacteria* and *Bacilli* was observed only in saliva. Previous 16S rRNA gene sequencing studies of the subgingival microbiome consistently identified the species *Actinomyces naeslundii*, *Actinomyces* sp. human oral taxon 171, *Rothia aeria*, *Rothia dentocariosa*, and *Streptococcus sanguinis* as health-associated taxa, since these taxa were replaced by pathogenic microbiota under periodontitis conditions (Abusleme et al., 2013; Hong et al., 2015; Curtis et al., 2020). Of note, these health-associated species are classified as the *Actinobacteria* or *Bacilli* class. Based on these results, we envisage that saliva may play an important role in maintaining the healthy oral microbiome. We also envisage that the salivary microbiome composition may be valuable to evaluate oral health status.

We examined the oral microbiome by using two independent cohorts of the TMM and found important microbial taxa for oral diseases. Our current results further suggest that in addition to its importance as a probe for oral diseases, oral microbiome analysis will emerge as a new approach for searching for therapeutic and preventive targets of systemic diseases.

## Data Availability Statement

The data presented in the study are deposited in jMorp (https://jmorp.megabank.tohoku.ac.jp/) and dbTMM (http://www.dist.megabank.tohoku.ac.jp/) release version 2.0.2.

## Ethics Statement

The studies involving human participants were reviewed and approved by the Ethics Committees of Tohoku University Graduate School of Medicine and by the Ethics Committees of Iwate Medical University. Written informed consent was provided by all of the participants. For participants with insufficient ability to understand the study protocol, we obtained informed consent from their guardians with approval by the ethics committees.

## Author Contributions

SS, RY, OT, AT, RS, and MY developed the concepts and designed the study. TT, MG, HM, JK, AH, SKuri, YS, NF, SKure, and AT conducted the cohort studies and collected the biospecimens and clinical information. RY and NM conducted biospecimen banking. SS, ID, and RY designed and performed the experiments. YA, TT, and KK performed the bioinformatics analysis. SS, YA, TT, MG, AT, and RS interpreted the results and prepared draft manuscript. All authors contributed to the article and approved the submitted version.

## Funding

This work was supported by Tohoku Medical Megabank Project from the Ministry of Education, Culture, Sports, Science and Technology (MEXT) and the Japan Agency for Medical Research and Development (AMED) under the grant numbers JP20km0105001 and JP20km0105002. It was also supported by the Advanced Genome Research and Bioinformatics Study to Facilitate Medical Innovation (GRIFIN) grant number JP20km0405203 and Facilitation of R&D Platform for AMED Genome Medicine Support conducted by AMED grant number JP20km0405001.

## Conflict of Interest

The authors declare that the research was conducted in the absence of any commercial or financial relationships that could be construed as a potential conflict of interest.
