# Supplementary material for: Oral Microbiome Analysis in Prospective Genome Cohort Studies of the Tohoku Medical Megabank Project
Source: Front Cell Infect Microbiol. 2021 Jan 29;10:604596. doi: 10.3389/fcimb.2020.604596 (PMC7878372; doi:10.3389/fcimb.2020.604596)
Supplement: Supplementary file 1 [file DataSheet_1.pdf]

Supplementary Material

Oral microbiome analysis in prospective genome cohort studies of the Tohoku Medical Megabank Project

Sakae Saito[1], Yuichi Aoki[1,2], Toru Tamahara[1,3], Maki Goto[1], Hiroyuki Matsui[1,3], Junko Kawashima[1], Inaho Danjoh[1,4], Atsushi Hozawa[1,4], Shinichi Kuriyama[1,4,5], Yoichi Suzuki[1,6], Nobuo Fuse[1,4], Shigeo Kure[1,4], Riu Yamashita[1,7], Osamu Tanabe[1,8], Naoko Minegishi[1,4,9], Kengo Kinoshita[1,2,9], Akito Tsuboi[1,3,4], Ritsuko Shimizu[1,4*], Masayuki Yamamoto[1,4,9*]

[1]Tohoku Medical Megabank Organization, Tohoku University, Sendai, Japan
[2]Graduate School of Information Sciences, Tohoku University, Sendai, Japan
[3]Graduate School of Dentistry, Tohoku University, Sendai, Japan
[4]Graduate School of Medicine, Tohoku University, Sendai, Japan
[5]International Research Institute of Disaster Science, Tohoku University, Sendai, Japan
[6]Department of Clinical Genetics, Ageo Central General Hospital, Ageo, Japan
[7]Exploratory Oncology Research & Clinical Trial Center, National Cancer Center, Kashiwa, Japan
[8]Biosample Research Center, Radiation Effects Research Foundation, Hiroshima, Japan
[9]Advanced Research Center for Innovations in Next-Generation Medicine, Tohoku University, Sendai, Japan

* **Correspondence:**
Ritsuko Shimizu; rshimizu@med.tohoku.ac.jp
Masayuki Yamamoto; masiyamamoto@med.tohoku.ac.jp

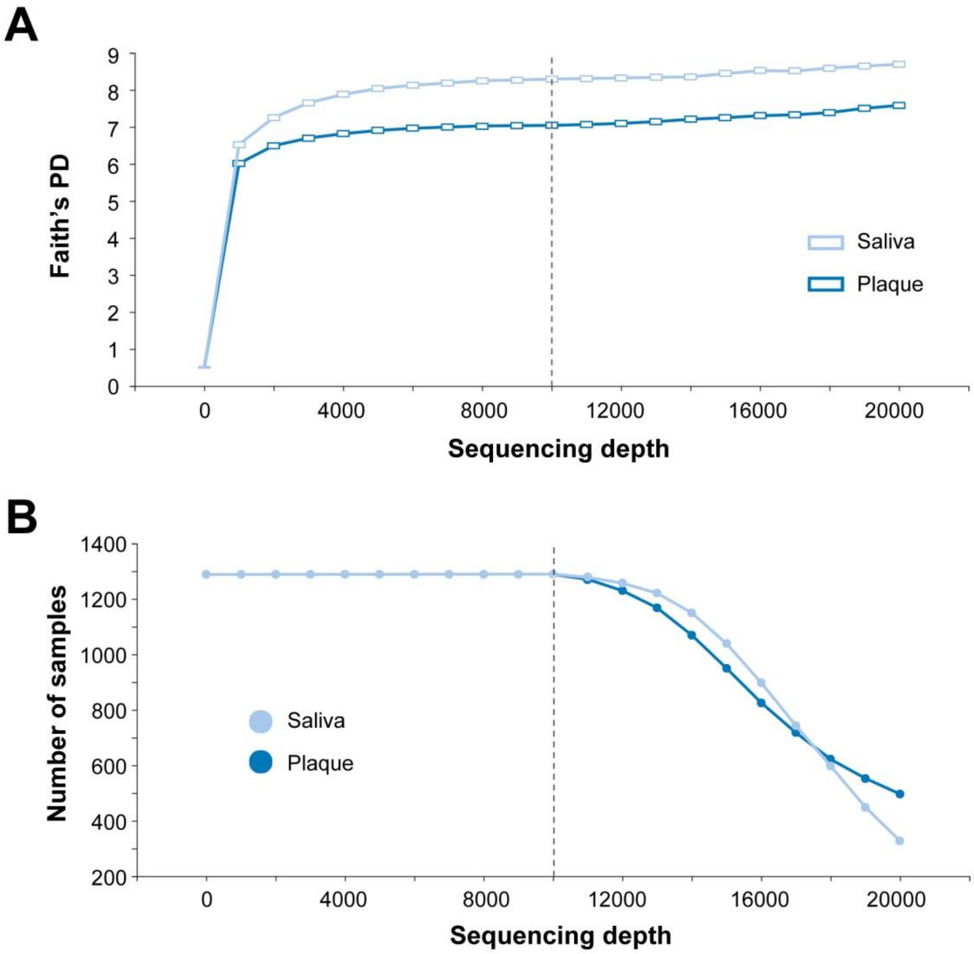

Supplementary Figure S1. The α-rarefaction analysis. (A) Rarefaction curves based on Faith's phylogenetic diversity (PD). **(B)** The curves of the sample numbers at the indicated rarefied sequencing depths. The colors indicate sample type: saliva (light blue) and plaque (blue). The vertical black dashed line indicates the 10,000-read rarefaction threshold. Data obtained from 1,349 participants in the TMM CommCohort Study and the TMM BirThree Cohort Study were analyzed.

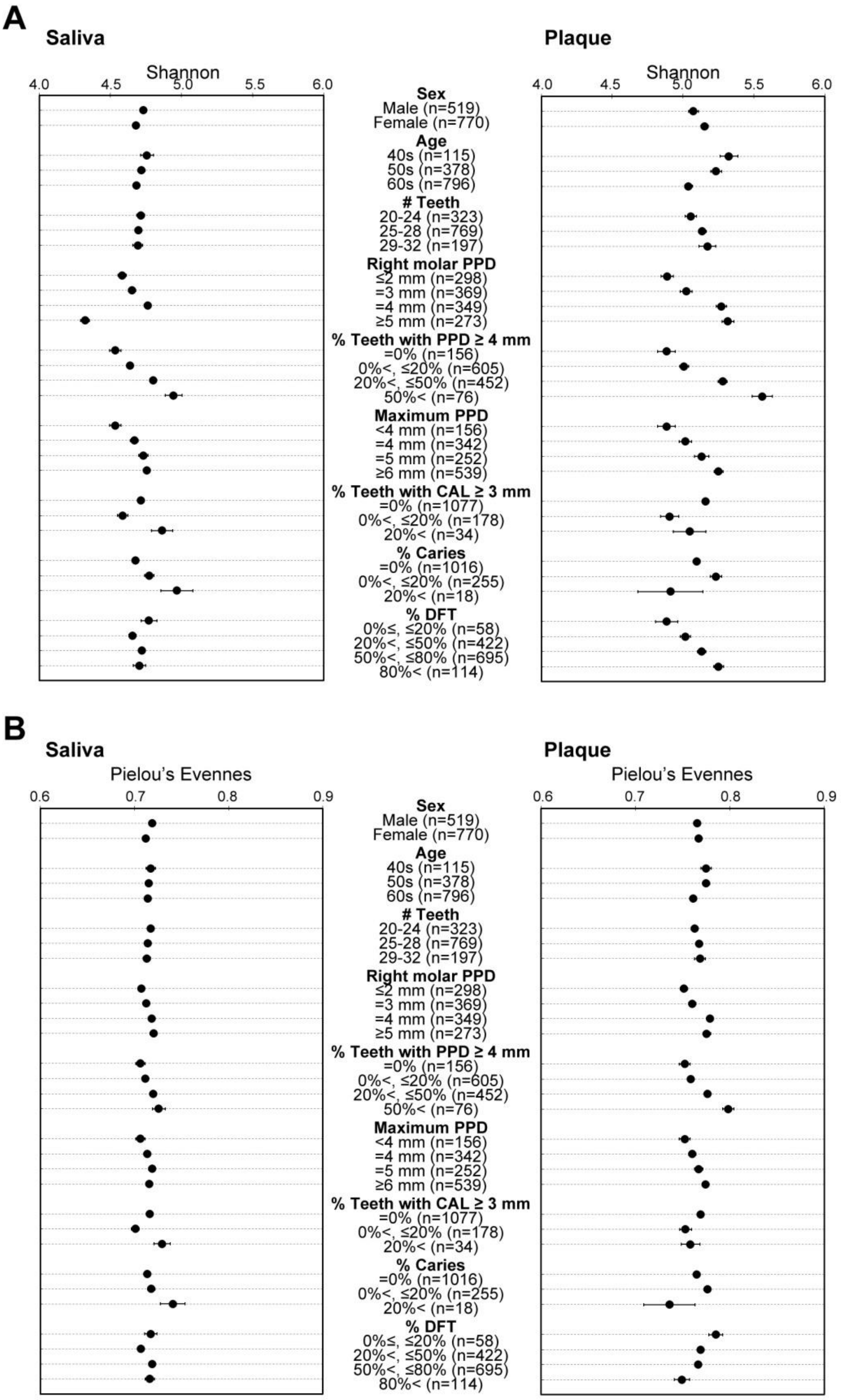

Supplementary Figure S2. The α-diversity of oral microbiota based on various general and dental conditions. (A) Shannon diversity index and **(B)** Pielou's evenness index of 1,289 participants in both the TMM CommCohort Study and the TMM BirThree Cohort Study based on 10,000 high-quality reads from both saliva and plaque samples. The dots indicate the means; the error bars indicate the standard errors (SEs). PPD; periodontal pocket depth, CAL; clinical attachment level, DFT; decayed and filled teeth.

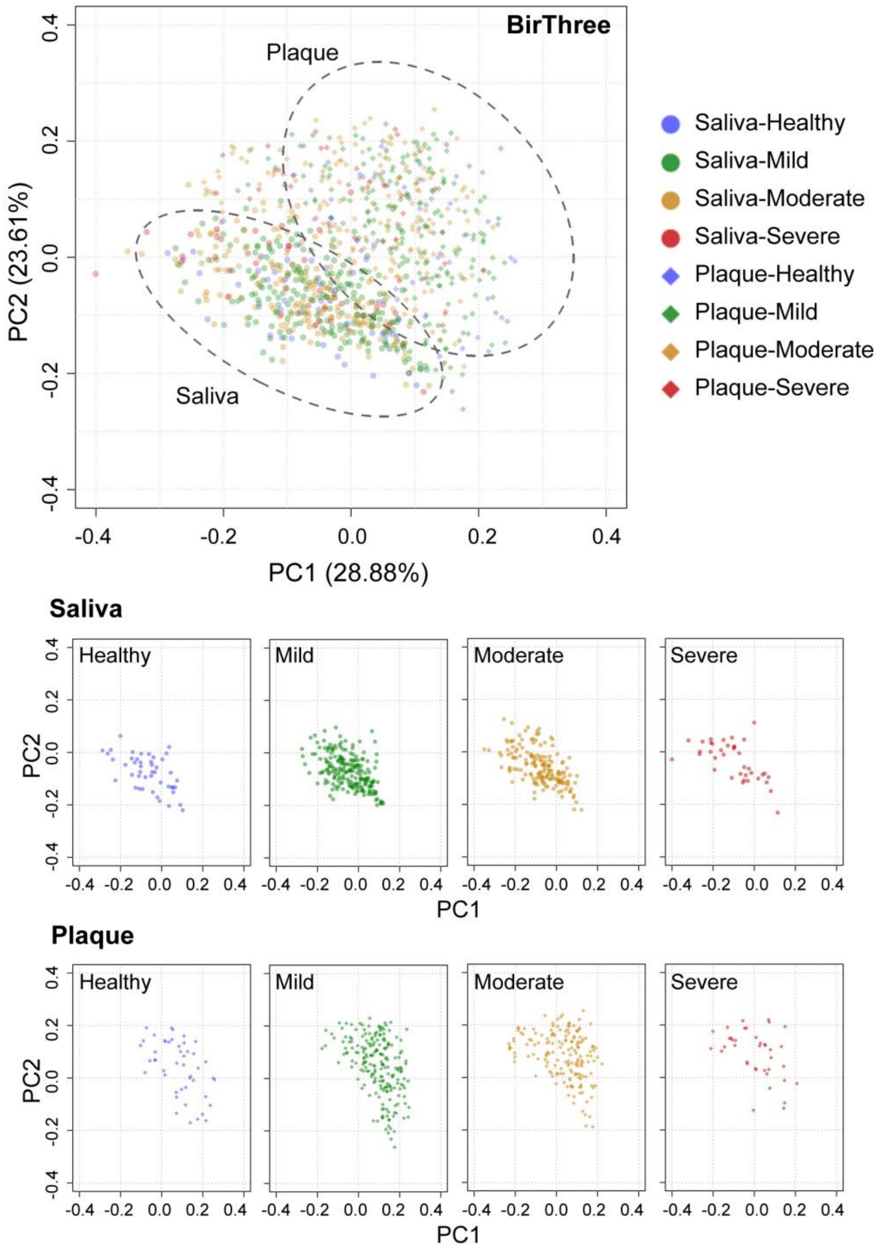

Supplementary Figure S3. PCoA plots of oral microbial communities in the TMM BirThree Cohort Study based on weighted UniFrac distance. Each dot represents the microbial community in the saliva (circles, n = 455) and plaque (diamonds, n = 455) of one individual. The dots in the top panel were separated into eight groups by sample type, which are shown at the bottom. The severity of periodontal disease was classified into four categories based on the ratio of teeth with deep pockets: healthy (0%, blue), mild (> 0% and ≤ 20%, green), moderate (> 20% and ≤ 50%, yellow), and severe (> 50%, red).

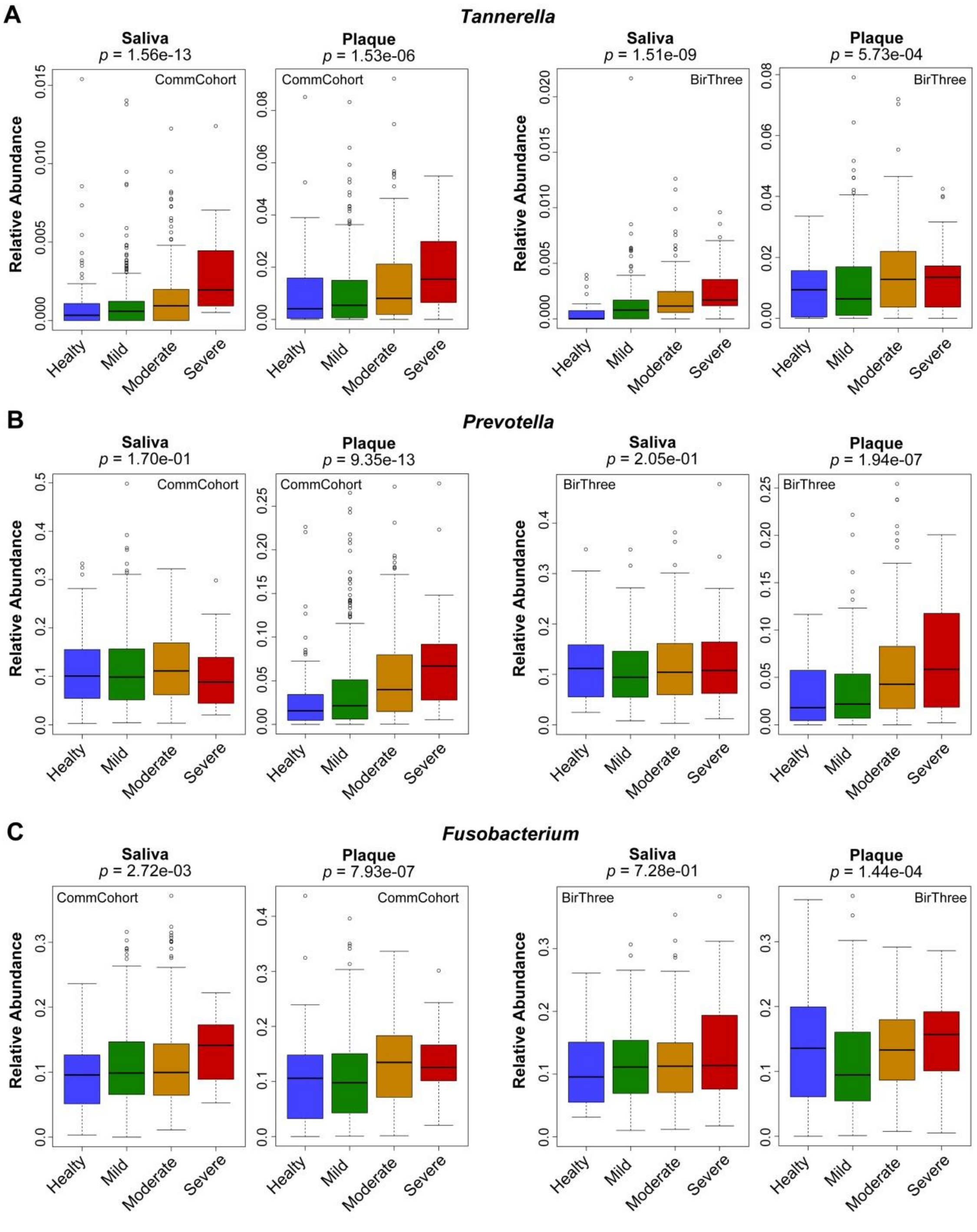

Supplementary Figure S4. Comparison of the relative microbial abundances of periodontal disease-causing bacteria at the genus level. Box plots showing the relative abundances of the genera *Tannerella* **(A)**, *Prevotella* **(B)**, and *Fusobacterium* **(C)** among the four periodontal disease severity categories in saliva and plaque. The significance of the differences was estimated by using logistic regression analysis.

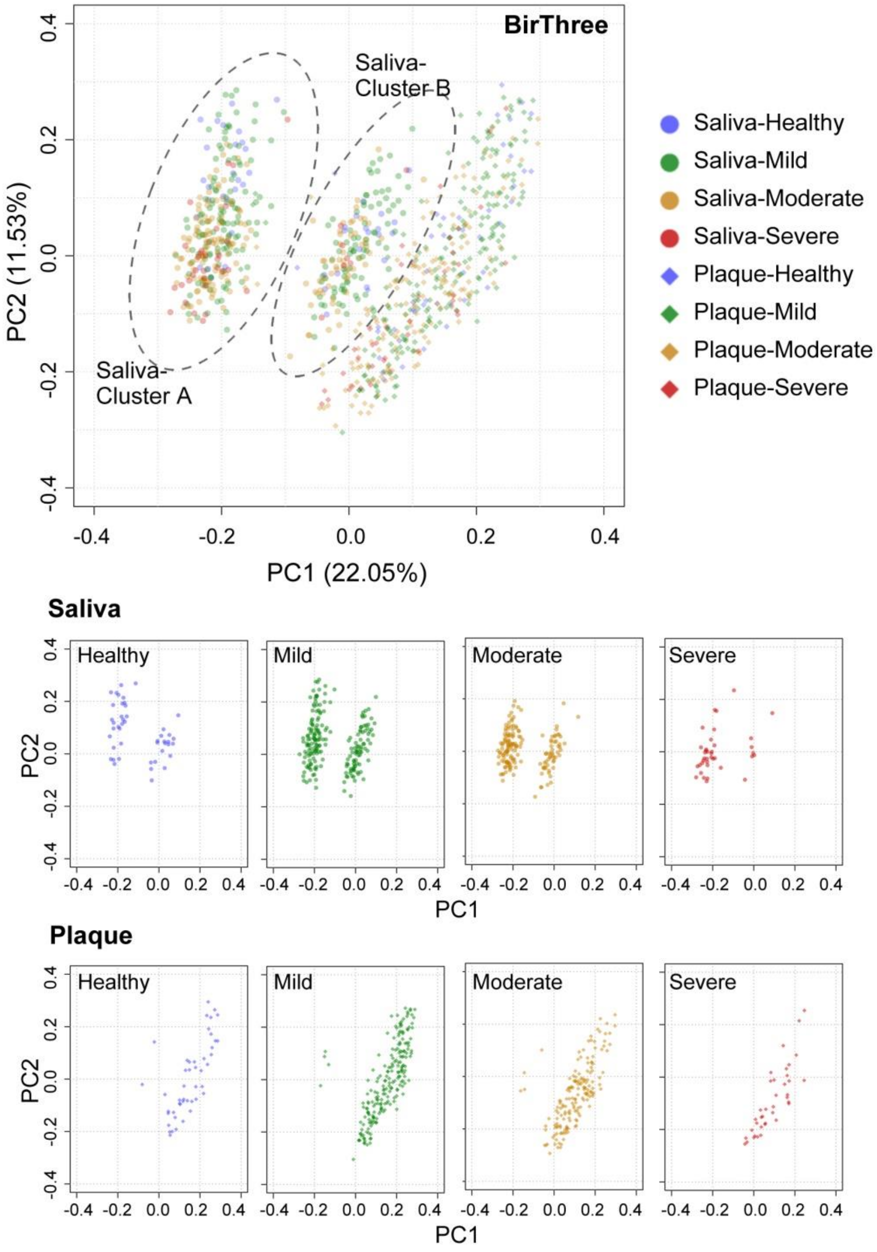

Supplementary Figure S5. PCoA plots of oral microbial communities in the TMM BirThree Cohort Study based on unweighted UniFrac distance. Each dot represents the microbial community in the saliva (circles, n = 455) and plaque (diamonds, n = 455) of one individual. The dots in the top panel were separated into eight groups by sample type, which are shown at the bottom. A distinct separation of the saliva samples that fall into two clusters (cluster A and cluster B) according to the PC1 axis is shown. The severity of periodontal disease was classified into four categories based on the ratio of teeth with deep pockets: healthy (0%, blue), mild (> 0% and ≤ 20%, green), moderate (> 20% and ≤ 50%, yellow), and severe (> 50%, red).

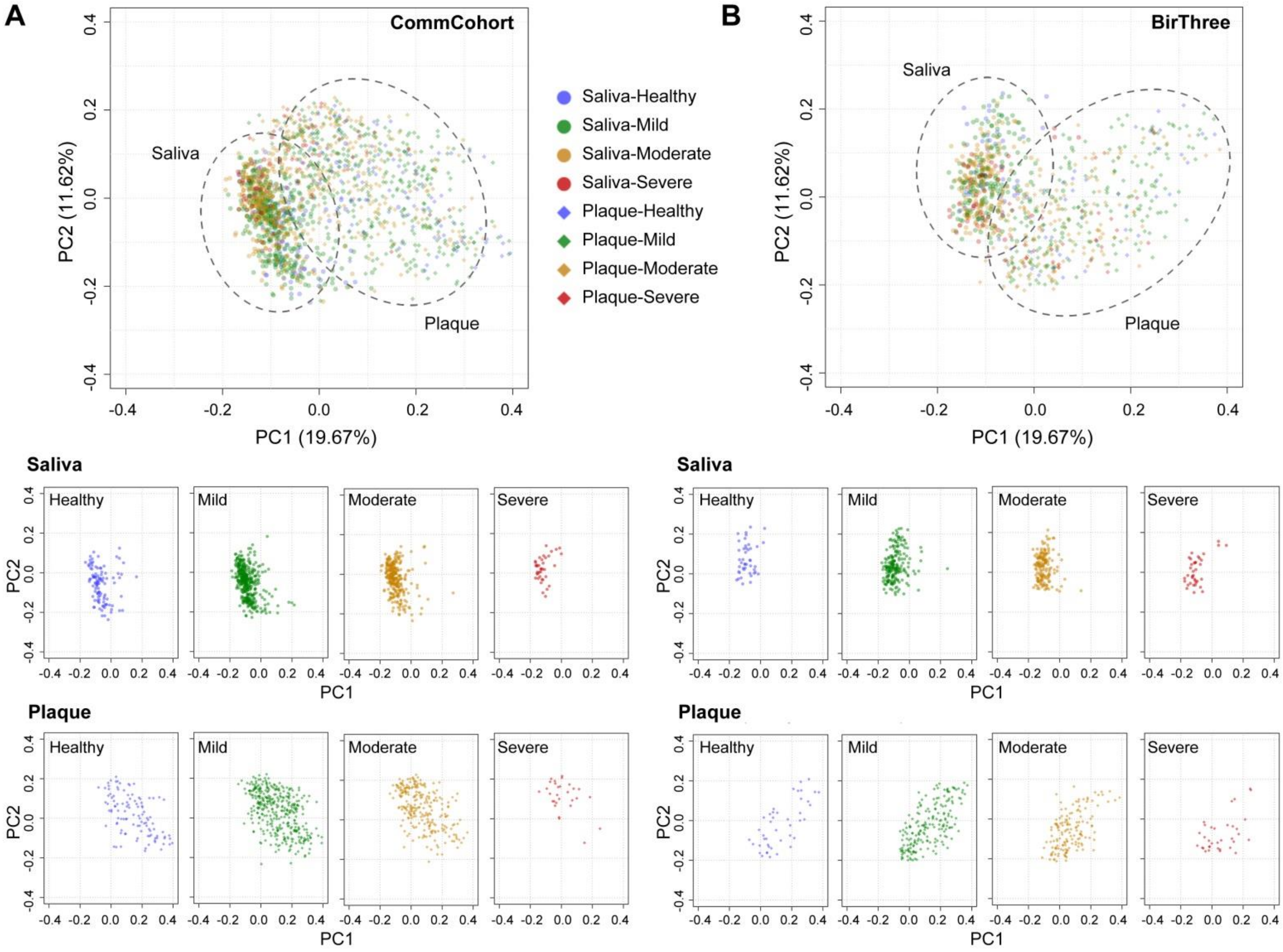

Supplementary Figure S6. PCoA plots of oral microbial communities excluding minor ASVs based on unweighted UniFrac distance. ASVs detected in less than 500 samples were defined as minor. **(A)** PCoA plots based on the unweighted UniFrac distances in the TMM CommCohort Study. Each dot represents the microbial community in the saliva (circle, n = 834) and plaque (diamond, n = 834) of one individual. **(B)** PCoA plots based on the unweighted UniFrac distances in the TMM BirThree Cohort Study. Each dot represents the microbial community in the saliva (circle, n = 455) and plaque (diamond, n = 455) of one individual. The dots in the top panel were separated into eight groups by sample type, which are shown at the bottom. The severity of periodontal disease was classified into four categories based on the ratio of teeth with deep pockets: healthy (0%, blue), mild (> 0% and ≤ 20%, green), moderate (> 20% and ≤ 50%, yellow), and severe (> 50%, red).

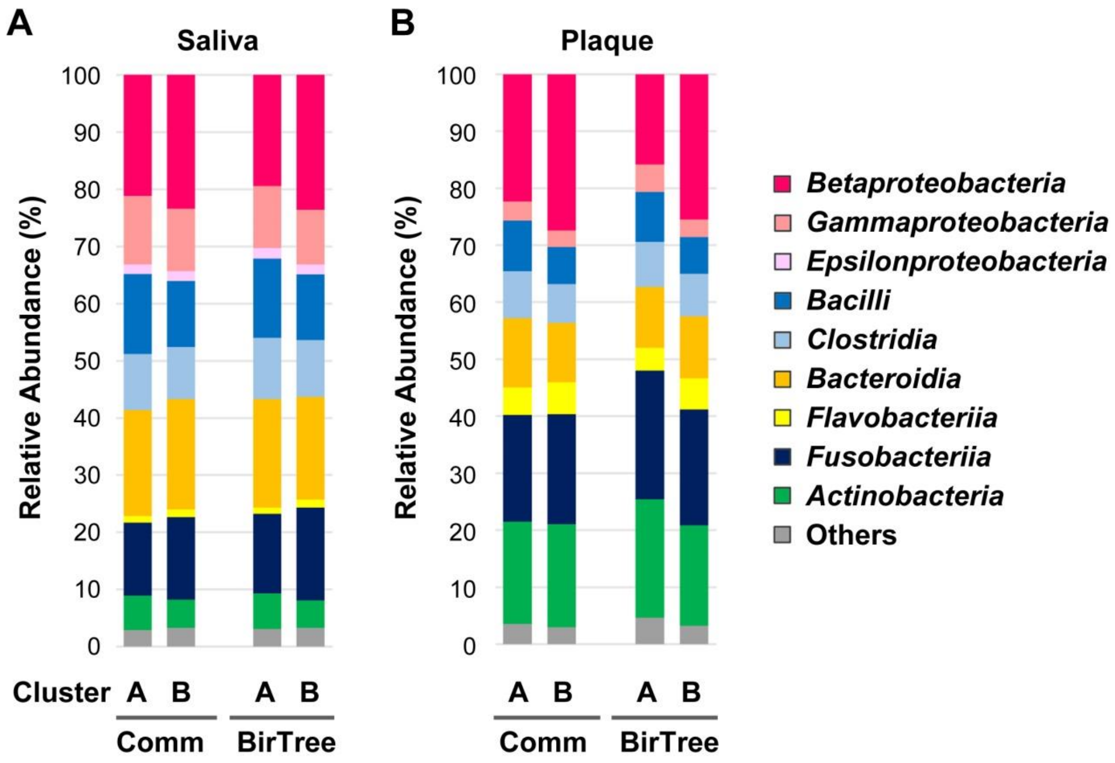

Supplementary Figure S7. Comparison of microbial composition in cluster A and B. Stacked bar plots showing the mean relative abundance of microbial taxa at the class level in saliva **(A)** and in plaque **(B)**. Data from the TMM CommCohort Study and the TMM BirThree Cohort Study were used for discovery and validation, respectively. The labels indicate the top 9 taxa at the class level, and the remaining classes were binned together.

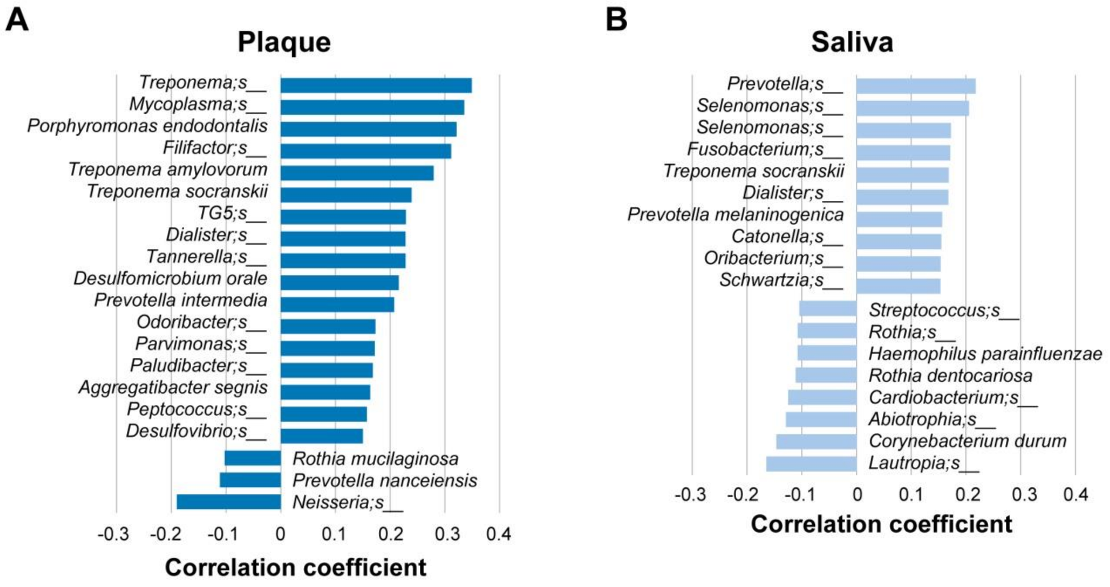

Supplementary Figure S8. Significant correlation between microbial taxa and periodontal disease severity. Bar plot indicating Pearson's correlation coefficients between the ratio of deep pockets (≥ 4 mm) and the relative microbial abundance in plaque **(A)** and saliva **(B)**. The microbial taxa (correlation coefficient ≥ 1.5 or ≤ -0.1) at the species level are arranged in descending order by correlation coefficient.
